# Quality of Life, Mental Health, and CPAP Compliance in Thai Patients with Obstructive Sleep Apnea during COVID-19 Pandemic

**DOI:** 10.1155/2024/1373299

**Published:** 2024-04-23

**Authors:** Kanyada Leelasittikul, Apiwat Pugongchai, Chatkarin Tepwimonpetkun, Narongkorn Saiphoklang

**Affiliations:** ^1^Medical Diagnostics Unit, Thammasat University Hospital, Khlong Nueng, Pathum Thani, Thailand; ^2^Sleep Center of Thammasat, Thammasat University Hospital, Khlong Nueng, Pathum Thani, Thailand; ^3^Department of Internal Medicine, Faculty of Medicine, Thammasat University, Khlong Nueng, Pathum Thani, Thailand

## Abstract

**Background:**

This study is aimed at determining the quality of life, mental health, and adherence to continuous positive airway pressure (CPAP) therapy for obstructive sleep apnea (OSA) among Thai OSA patients during the coronavirus disease 2019 (COVID-19) pandemic as this data has been lacking.

**Methods:**

A cross-sectional study was conducted at a university hospital between September 2021 and April 2022. OSA patients aged 18 years or older who required home CPAP treatment were included. Sleep Apnea Quality of Life Index (SAQLI) and Depression Anxiety Stress Scales-21 (DASS-21) were used to assess quality of life and mental health, respectively.

**Results:**

A total of 142 participants (62% male) were included, with a mean age of 54.4 ± 14.7 years and a body mass index of 29.9 ± 6.8 kg/m^2^. Polysomnographic data showed a mean apnea-hypopnea index of 48.0 ± 32.4 events/hour and a mean lowest oxygen saturation of 79.2 ± 12.2%. Severe OSA was observed in 66.9%. CPAP compliance was reported in 50.7%. The SAQLI score was 2.32 ± 1.12. Depression, anxiety, and stress scores in DASS-21 were 2.89 ± 3.31, 3.94 ± 3.67, and 4.82 ± 4.00, respectively. Compared to the CPAP compliance group, the CPAP noncompliance group had higher daily activity scores in SAQLI (2.98 ± 1.25 vs. 2.45 ± 1.33, *P* = 0.015).

**Conclusions:**

The quality of life for Thai OSA patients during the COVID-19 era was moderate degree. Poor CPAP compliance was significantly associated with limited daily activity. Enhancing CPAP compliance could improve the quality of life in these patients. This trial is registered with TCTR20211104004.

## 1. Introduction

The coronavirus disease 2019 (COVID-19) pandemic is a serious worldwide respiratory tract infection. It affects people's health, the economy, and society. The effects have led to poor quality of life and impacted mental health [1]. Patients with COVID-19 were required to undergo home isolation or hospital admission. City lockdowns adversely affected the quality of life and mental health, resulting in decreased daily activity and increased anxiety, stress, and depression. These effects were observed in COVID-19 patients, other patients with preexisting conditions, the general population, and healthcare personnel [2–4].

Obstructive sleep apnea (OSA) is a chronic respiratory disease characterized by partial or complete collapse of the upper airway during sleep. This condition leads to repeated cessation of breathing or decreased airflow, sleep fragmentation, and episodes of oxygen desaturation, resulting in excessive daytime sleepiness, irritability, attention deficit hyperactivity disorder, poor work performance, and poor quality of life [5, 6]. Diagnosis of OSA is based on clinical symptoms and risks, and it is confirmed through polysomnography (PSG) [7].

Continuous positive airway pressure (CPAP) is a highly effective treatment for OSA patients. Good CPAP compliance, defined as using CPAP at least 4 hours per night on at least 70% of nights [8], is associated with better clinical outcomes for OSA patients [6, 9].

However, the COVID-19 pandemic has had a remarkable effect on several aspects of the general population, and its impact is probably greater on individuals suffering from chronic illnesses, especially on their quality of life and mental health. Data on these issues in OSA patients have been limited. Therefore, this study is aimed at determining the quality of life, mental health, and CPAP compliance among Thai OSA patients during the COVID-19 pandemic.

## 2. Materials and Methods

### 2.1. Study Design and Participants

This cross-sectional study was conducted at Thammasat University Hospital in Thailand between September 2021 and April 2022. This timeframe did not coincide with the COVID-19 lockdown period (April to May 2020), allowing Thai people to travel freely. However, preventive measures such as social distancing, mask-wearing, hand hygiene, and COVID-19 testing were still necessary. Subjects aged 18 years or older with OSA requiring CPAP treatment during a follow-up visit were included. Exclusion criteria were the inability to download CPAP usage data due to issues with the CPAP machine or software, communication challenges, and psychiatric or neurological disorders. Demographic information, polysomnographic data, quality of life, mental health data, and CPAP compliance were recorded.

Ethics approval was obtained from the Ethics Committee of the Faculty of Medicine, Thammasat University (IRB No. MTU-EC-OO-6-215/64; COA No. 227/2021), in compliance with the Declaration of Helsinki, the Belmont Report, CIOMS Guidelines, and the International Council for Harmonisation Guideline for Good Clinical Practice (ICH-GCP). All methods were performed in accordance with these guidelines and regulations. All participants provided written informed consent.

### 2.2. Procedures and Outcomes

All participants completed data on demographics, body mass index, occupation, income, level of education, comorbidity, the Thai version of the Epworth Sleepiness Scale (ESS) [10], quality of life, and mental health. Clinical data were collected using a questionnaire completed by the patients themselves and subsequently reviewed by a study coordinator. The Sleep Apnea Quality of Life Index (SAQLI) was used to assess the quality of life and has been validated for evaluating the quality of life in Thai OSA patients, demonstrating a Cronbach's alpha coefficient and external reliability of 0.90 and 0.93, respectively [11]. The SAQLI covers four domains: daily activity, social interaction, emotion, and symptoms. A high score indicates a poor quality of life.

Mental health was assessed using the Depression Anxiety Stress Scale-21 (DASS-21), which demonstrated Cronbach's alpha coefficients of 0.89 for depression, 0.83 for anxiety, and 0.92 for stress [12]. The DASS-21 comprises 21 items evaluating depression, anxiety, and stress, with a higher score indicating poorer mental health. Depression was defined as DASS-21 depression scores ≥ 5 (mild 5-6, moderate 7-10, severe 11-13, and extremely severe ≥ 14). Anxiety was defined as DASS-21 anxiety scores ≥ 4 (mild 4-5, moderate 6-7, severe 8-9, and extremely severe ≥ 10). Stress was defined as DASS-21 stress scores ≥ 8 (mild 8-9, moderate 10-12, severe 13-16, and extremely severe ≥ 17).

PSG data, including the apnea-hypopnea index (AHI) and lowest oxygen (O_2_) saturation, reflecting the severity of obstructive sleep apnea (OSA), were collected from the PSG laboratory at Thammasat University Hospital. Additionally, CPAP compliance was assessed using records generated by the machine's software. All data were collected from a single visit during the COVID-19 pandemic. The severity of OSA was classified using AHI values: AHI < 5, no OSA; AHI 5-14, mild OSA; AHI 15-29, moderate OSA; and AHI ≥ 30, severe OSA [7]. CPAP compliance was defined as usage for a minimum of 4 hours per night on at least 70% of nights [8].

### 2.3. Statistical Analysis

Based on a previous study in the year 2017 [13], stress scores assessed by DASS-21 in patients with untreated OSA in the pre-COVID-19 era were 6.74 ± 7.00. We hypothesized that our study would have a 2-point higher score than that of the previous study. The sample size was calculated using 90% power and a 5% type. Thus, the calculated sample size would be 142.

Data are presented as numbers (%) and mean ± standard deviation (SD). The chi-squared test was used to compare categorical variables between the CPAP compliance and noncompliance groups. Student's *t*-test was employed to compare continuous variables between the two groups. Two-tailed *P* values of less than 0.05 were considered statistically significant. All data analyses were performed using SPSS version 26.0 software (IBM Corp., Armonk, NY, USA).

## 3. Results

### 3.1. Participants

One hundred forty-two patients were included in the study. The mean age was 54.4 ± 14.7 years. 62% were male. Body mass index was 29.9 ± 6.8 kg/m^2^. Comorbid diseases included hypertension (64.1%), dyslipidemia (47.2%), and diabetes (28.9%). PSG data showed a mean AHI of 48.0 ± 32.4 events/hour and a mean lowest O_2_ saturation of 79.2 ± 12.2 percent. Most patients had severe OSA (66.9%), and good compliance was observed in 50.7% (Table 1).

### 3.2. Quality of Life and Mental Health

The mean total score of SAQLI was 2.32 ± 1.12, indicating a moderate level of quality of life. Subdomain scores for daily activity, social interaction, emotion, and symptoms in SAQLI were 2.71 ± 1.31, 2.30 ± 1.24, 1.80 ± 1.26, and 2.31 ± 1.53, respectively. In comparison to the CPAP compliance group, the CPAP noncompliance group had significantly higher daily activity scores (2.98 ± 1.25 vs. 2.45 ± 1.33, *P* = 0.015) (Table 2).

Scores of depression, anxiety, and stress in DASS-21 were 2.89 ± 3.31, 3.94 ± 3.67, and 4.82 ± 4.00, respectively, indicating normal mental health. The CPAP compliance group showed no statistically significant difference in all category scores of DASS-21 compared to the noncompliance group (Table 3).

## 4. Discussion

This study evaluates the association between quality of life, mental health, and CPAP compliance in OSA patients during the COVID-19 era. The patients demonstrated a moderate degree of quality of life, and CPAP noncompliance was significantly associated with limited daily activities. In a corresponding study, Maierean et al. demonstrated that OSA patients experienced a higher level of anxiety, changes in sleep schedule, and weight gain due to factors such as job loss, isolation, and emotional changes. These factors influenced mental health during the pandemic [14].

The rate of sleep disturbance increased from 54% to 66% in OSA patients and from 29% to 40% in the control group during the COVID-19 pandemic, as reported by Spicuzza et al. However, there was no significant difference in the percentage of depression between OSA patients and controls (61% vs. 65%) [15]. A review by Gill et al. found that adherence to CPAP therapy may have improved, and the use of remote consultations and telemonitoring increased during the COVID-19 pandemic [16]. A study by Demirovic et al. demonstrated that CPAP adherence improved during the COVID-19 lockdown in severe OSA patients [17]. Telemonitoring for CPAP use was effective at improving adherence to OSA treatment during the pandemic, as demonstrated in a study by Gassama et al. [18]. Our results showed that the CPAP compliance rate was moderate, even without remote consultations and telemonitoring.

OSA patients who adhered to CPAP therapy were less likely to experience a severe course of COVID-19 or death compared to those who were noncompliant with the therapy [19]. OSA patients exhibited greater adherence to CPAP treatment during the COVID-19 pandemic compared to the prepandemic period [20, 21]. This might be attributed to home isolation during the pandemic, resulting in improved sleep and continuous CPAP compliance. However, a study by Michalek-Zrabkowska et al. reported that the COVID-19 pandemic had no significant impact on CPAP therapy parameters and adherence in OSA patients [22]. Additionally, a large study involving 8,477 OSA patients by Bertelli et al. demonstrated that the COVID-19 pandemic had a clinically irrelevant effect on CPAP adherence for OSA treatment [23]. Our study showed that CPAP compliance was 50%, consistent with a study by Rotenberg et al., which demonstrated CPAP compliance around 60% in normal situations [24]. Similar to the study by Tepwimonpetkun et al. in the year 2022, their findings indicated that there was no statistically significant difference in CPAP compliance between the prepandemic and pandemic periods [25].

Regarding the relationship between CPAP compliance and mental health during the COVID-19 pandemic, our study revealed no significant difference in mental health levels between patients with good and poor CPAP compliance. This could be attributed to the availability of vaccines, which help prevent the disease or reduce its severity, as well as the population's gradual adaptation to the pandemic situation.

Our study showed that patients with CPAP noncompliance had slightly higher mental health scores compared to patients with good compliance, indicating poorer mental health. This result is consistent with a study by Sunbul et al. in the year 2022, which found that among patients with poor CPAP compliance, there was a significantly higher prevalence of anxiety symptoms compared to patients with good CPAP compliance [26]. It was observed that increased anxiety was also related to poor sleep quality [27]. Furthermore, the group with poor sleep quality had a 2.14 times higher risk of developing depression in the year 2019 and a 2.26 times higher risk in the year 2020. Additionally, it had a higher risk of experiencing high levels of stress compared to the group with good sleep quality [28].

This study has certain limitations. Firstly, our study lacked data from the prepandemic period for comparison with the pandemic period; therefore, we cannot determine the difference between the two periods. Secondly, there was no control group for comparison with our study group; hence, we cannot distinguish differences from the general population.

## 5. Conclusions

The overall quality of life for Thai patients with OSA during the COVID-19 pandemic was moderate degree. Patients with poor CPAP compliance showed a correlation with limited daily activity compared to patients with good CPAP compliance. However, the mental health levels of OSA patients during the COVID-19 outbreak remained within normal ranges. Increasing CPAP compliance may contribute to improving the quality of life for patients with OSA.

## Figures and Tables

**Table 1:** Baseline characteristics of patients with obstructive sleep apnea.

Characteristics	Data (*n* = 142)
Age (years)	54.4 ± 14.7
Male/female	88 (62.0)/54 (38.0)
BMI (kg/m^2^)	29.9 ± 6.8
ESS (points)	9.8 ± 5.3
Occupation	
Government	55 (38.7)
Nongovernment	75 (52.8)
Unemployed	12 (8.5)
Income (USD per month)	
<450	51 (35.9)
≥450	91 (64.1)
Level of education	
Associate degree/high school or lower	44 (31.0)
Bachelor's degree or higher	98 (69.0)
Comorbidity	
Hypertension	91 (64.1)
Dyslipidemia	67 (47.2)
Allergic rhinitis	44 (31.0)
Diabetes	41 (28.9)
Obesity	39 (27.5)
Polysomnographic data	
AHI (events/hour)	48.0 ± 32.4
Lowest SpO_2_ (%)	79.2 ± 12.2
OSA severity	
Mild	16 (11.3)
Moderate	29 (20.4)
Severe	95 (66.9)
Home CPAP data	
CPAP use (hours/night)	5.07 ± 1.87
CPAP compliance^∗^	70 (50.7)

Data are shown as *n* (%) or mean ± SD. ∗ is defined as usage greater or equal to 4 hours per night on 70% of nights. AHI = apnea-hypopnea index; BMI = body mass index; CPAP = continuous positive airway pressure; ESS = Epworth Sleepiness Scale; OSA = obstructive sleep apnea; SpO_2_ = peripheral oxygen saturation; USD = the United States dollar.

**Table 2:** Comparison of the Sleep Apnea Quality of Life Index between CPAP compliance and noncompliance in OSA patients.

Variables	Total (*n* = 142)	CPAP noncompliance (*n* = 70)	CPAP compliance (*n* = 72)	*P* value
Total score	2.32 ± 1.12	2.50 ± 1.08	2.15 ± 1.13	0.062
Daily activity	2.71 ± 1.31	2.98 ± 1.25	2.45 ± 1.33	0.015
Social interaction	2.30 ± 1.24	2.44 ± 1.23	2.21 ± 1.25	0.268
Emotion	1.80 ± 1.26	1.93 ± 1.26	1.67 ± 1.26	0.216
Symptom	2.31 ± 1.53	2.48 ± 1.52	2.14 ± 1.53	0.197

Data are shown as mean ± SD. CPAP compliance is defined as usage greater than or equal to 4 hours per night on 70% of nights. CPAP = continuous positive airway pressure; OSA = obstructive sleep apnea.

**Table 3:** Comparison of the Depression Anxiety Stress Scale-21 between CPAP compliance and noncompliance in OSA patients.

Variables	Total (*n* = 142)	CPAP noncompliance (*n* = 70)	CPAP compliance (*n* = 72)	*P* value
DASS-21, score	11.64 ± 10.19	11.89 ± 10.34	11.40 ± 10.11	0.779
Depression	2.89 ± 3.31	2.97 ± 3.31	2.82 ± 3.34	0.786
Anxiety	3.94 ± 3.67	4.06 ± 3.83	3.82 ± 3.53	0.701
Stress	4.82 ± 4.00	4.86 ± 4.06	4.79 ± 3.88	0.922
DASS-21 subgroup				
Depression	34 (23.9)	18 (25.7)	16 (22.2)	0.626
Anxiety	62 (43.7)	32 (45.7)	30 (41.7)	0.627
Stress	29 (20.4)	13 (18.6)	16 (22.2)	0.590
DASS-21 subscale				
Depression				0.116
Mild	15 (10.6)	9 (12.9)	6 (8.3)	
Moderate	13 (9.2)	5 (7.1)	8 (11.1)	
Severe	4 (2.8)	4 (5.7)	0 (0.0)	
Extremely severe	2 (1.4)	0 (0.0)	2 (2.8)	
Anxiety				0.914
Mild	25 (17.6)	12 (17.1)	14 (19.4)	
Moderate	20 (14.1)	11 (15.7)	9 (12.5)	
Severe	6 (4.2)	2 (2.9)	3 (4.2)	
Extremely severe	11 (7.7)	7 (10.0)	5 (6.9)	
Stress				0.432
Mild	12 (8.5)	5 (7.1)	7 (9.7)	
Moderate	8 (5.6)	2 (2.9)	6 (8.3)	
Severe	8 (5.6)	5 (7.1)	3 (4.2)	
Extremely severe	1 (0.7)	1 (1.4)	0 (0.0)	

Data are shown as *n* (%) or mean ± SD. CPAP compliance is defined as usage greater than or equal to 4 hours per night on 70% of nights. Depression is defined as DASS-21 depression score ≥ 5 points (mild = 5‐6, moderate = 7‐10, severe = 11‐13, and extremely severe ≥ 14). Anxiety is defined as DASS-21 anxiety score ≥ 4 points (mild = 4‐5, moderate = 6‐7, severe = 8‐9, and extremely severe ≥ 10). Stress is defined as DASS-21 stress score ≥ 8 points (mild = 8‐9, moderate = 10‐12, severe = 13‐16, and extremely severe ≥ 17). CPAP = continuous positive airway pressure; DASS-21 = Depression Anxiety Stress Scale-21; OSA = obstructive sleep apnea.

## Data Availability

The data supporting the results of this study are available within the article. Underlying data are shared on Figshare (10.6084/m9.figshare.24886644.v1). Data are available under the terms of the Creative Commons Attribution 4.0 International license (CC-BY 4.0).
